# Adaptive Genetic Divergence along Narrow Environmental Gradients in Four Stream Insects

**DOI:** 10.1371/journal.pone.0093055

**Published:** 2014-03-28

**Authors:** Kozo Watanabe, So Kazama, Tatsuo Omura, Michael T. Monaghan

**Affiliations:** 1 Leibniz-Institute of Freshwater Ecology and Inland Fisheries (IGB), Müggelseedamm 301, Berlin, Germany; 2 Present address: Department of Civil and Environmental Engineering, Ehime University, Bunkyo-cho 3, Matsuyama, Japan; 3 Department of Civil and Environmental Engineering, Tohoku University, Aoba-yama 6-6-06, Sendai, Japan; 4 New Industry Creation Hatchery Center (NIChe), Tohoku University, Aoba-yama 6-6-04, Sendai, Japan; University of Massachusetts, United States of America

## Abstract

A central question linking ecology with evolutionary biology is how environmental heterogeneity can drive adaptive genetic divergence among populations. We examined adaptive divergence of four stream insects from six adjacent catchments in Japan by combining field measures of habitat and resource components with genome scans of non-neutral Amplified Fragment Length Polymorphism (AFLP) loci. Neutral genetic variation was used to measure gene flow and non-neutral genetic variation was used to test for adaptive divergence. We identified the environmental characteristics contributing to divergence by comparing genetic distances at non-neutral loci between sites with Euclidean distances for each of 15 environmental variables. Comparisons were made using partial Mantel tests to control for geographic distance. In all four species, we found strong evidence for non-neutral divergence along environmental gradients at between 6 and 21 loci per species. The relative contribution of these environmental variables to each species' ecological niche was quantified as the specialization index, *S*, based on ecological data. In each species, the variable most significantly correlated with genetic distance at non-neutral loci was the same variable along which each species was most narrowly distributed (i.e., highest *S*). These were gradients of elevation (two species), chlorophyll-a, and ammonia-nitrogen. This adaptive divergence occurred in the face of ongoing gene flow (*F*_st_ = 0.01–0.04), indicating that selection was strong enough to overcome homogenization at the landscape scale. Our results suggest that adaptive divergence is pronounced, occurs along different environmental gradients for different species, and may consistently occur along the narrowest components of species' niche.

## Introduction

Adaptive genetic divergence among populations within species is an evolutionary process on the way towards ecological speciation [1] and is thought to be an important driver of the formation and maintenance of biodiversity [2]. As the potential causes of adaptive divergence, evolutionary biologists pointed to complex mechanisms that involve multiple drivers such as divergent natural selection against migrants ([3]), secondary contact following allopatric isolation (e.g., [4]), interspecific competition [5], and genetic linkage between quantitative trait loci (QTLs) (e.g., [6]).

There is experimental and descriptive evidence for adaptive divergence across a range of taxa and environments (e.g., [7]–[9]). Studies that investigate the genetic basis of adaptation often use candidate gene and QTL approaches [10]–[13]. Unfortunately these molecular genetic tools are largely limited to model organisms and well characterized genes, making it difficult to apply the approach to natural populations or to look for general patterns across multiple species. Genome scans allow for the study of non-model organisms and a number of studies have reported evidence for genetic adaptation, where changes in allele frequencies at putatively adaptive loci are correlated with environmental changes across the species' range (e.g., [14]–[17]). Such studies provide strong evidence for adaptive divergence among populations at one or more parts of the genome but are often limited to single species and a few environmental parameters. The result is that it is difficult to make any generalizations across taxa or environmental gradients in natural populations. Natural populations are subject to many selective forces simultaneously, and several environmental factors may be responsible for the observed genetic divergence.

An alternative approach is to study several co-occurring species in nature and multiple components of their environment and to look for general patterns of adaptive divergence (i.e. along similar environmental gradients). By screening large numbers of candidate loci and individuals [18]–[22], statistical methods can be used to identify loci that are either under direct selection or are linked to loci under selection (“outlier” loci; reviewed by [15]). Compared to loci that are neutral, loci influenced by directional selection should show more genetic differentiation, and loci subject to balancing selection should show less genetic differentiation. In practice, these statistical methods identify loci with *F*_ST_ values that are significantly different from those expected under neutral conditions.

Here we examined adaptive divergence in four species of aquatic insect from six adjacent stream catchments in northeastern Honshu Island, Japan. The species differ in their local distribution and thus are likely to have different ecological niches. Because most aquatic insect dispersal occurs in the adult stage, the distribution of aquatic immatures is primarily determined by local environmental characteristics [23]. There were three objectives in this study. The first was to measure the extent of adaptive divergence in natural populations. We genotyped individuals using Amplified Fragment Length Polymorphism (AFLP) loci and identified loci under selection (“outlier loci”) using locus-specific tests of genetic differentiation among sampling sites. The second objective was to examine the link between environmental heterogeneity and any observed non-neutral genetic divergence. We first measured 15 habitat variables at each location, and identified those characteristics most limiting species distributions in the study area using the ecological specialization index (*S*) [24]. We also identified environmental variables that are most likely to contribute to adaptive divergence for each species using Mantel tests, and compered the variables with those identified by the specialization index analyses. The third objective was to test whether the patterns across the four species were consistent.

## Methods

### Ethics statement

We did not require ethical approval to conduct this study as we did not handle or collect samples of any vertebrates. We received permissions to access study sites and to collect samples from the Ministry of Land, Infrastructure, Transport and Tourism (MLIT) of Japan (#07052442).

### Study area and species

**Figure 1:**
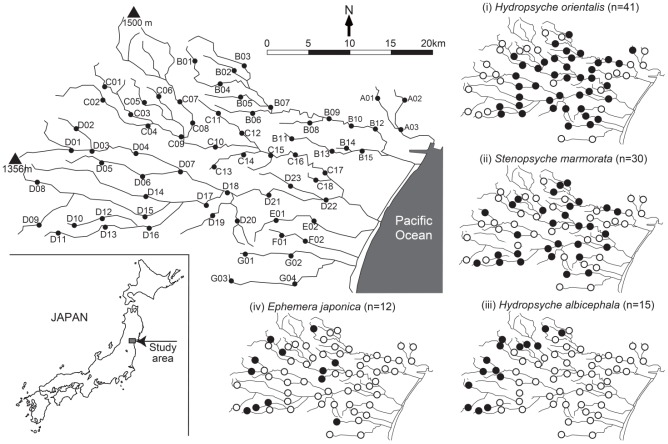
Map of the 62 study sites in Miyagi Prefecture, northeast Honshu, Japan. The four small panels (i–iv) show the presence (filled) and absence (open) of each species based on ecological surveys in summer (July) and autumn (November) 2006. The codes reflects the 6 different catchments.

The study was carried out in six adjacent stream catchments in Miyagi Prefecture, Japan (*ca* 120 sq km) (Fig. 1). Streams in the area are characterized by high environmental heterogeneity along short and steep corridors. The region is largely mountainous or hilly and forested with beech (*Fagus*) and cedar (*Cryptomeria*). Lowland areas are used for agriculture (13% of total study area, primarily rice paddy) or are a mix of residential and commercial areas (11%). Study sites (*n* = 62) were chosen to represent the wide range of physical and chemical habitat characteristics in the region.

The four species examined are typical of high-gradient, fast-flowing streams in Japan. Three were caddisflies (Trichoptera), *Hydropsyche orientalis* (Ho), *Stenopsyche marmorata* (Sm), and *Hydropsyche albicephela* (Ha); and one was a mayfly (Ephemeroptera), *Ephemera japonica* (Ej). Individuals of each species live in the water for up to one year before emergence, reproduction, and aerial dispersal. As aquatic larvae, they feed primarily on fine (<1 mm diameter) particulate organic matter that is either transported in the water column (Ho, Sm and Ha) or deposited on the stream bed (Ej). Sampling was conducted in July and November (summer and autumn) 2006. At each site, two person-hours were spent collecting larval individuals of as many of the target species as possible using a kick net (250 μm mesh) along a 300-m stream reach. If more than one individual was found in either July or November the species was considered present at the site. Samples were preserved in 99% ethanol in the field, returned to the laboratory and identified to species under a stereomicroscope (150 x).

### Environmental variables

We measured 31 biological, physical, and chemical characteristics of the habitat known to influence the distribution of larval insects in streams [23]. These included both resource (e.g., organic matter transported in the water column) and habitat (e.g., stream width) variables. Data were collected from each study site using standard methods in stream ecological surveys (e.g., [25], [26]). Using average values of July and November measurements, 15 of these variables were chosen for further analysis by eliminating variables that were correlated (Table S1 in File S1).

Of the 15 variables, altitude was measured using an altimeter at the center of each site. Stream order was determined using a 1∶25000 map. Width of the stream channel was measured at 10 randomly selected cross-sections using a tape measure. Water velocity was measured at 20 randomly selected points in riffles at 0.2 × water depth from the surface using an electromagnetic current meter. Substrate (gravel) size was calculated as the mean of the longest diameter of 36 pieces of gravel at grid points located in a haphazardly selected 1-sq m area of the stream bottom in a riffle. Benthic (on the stream bottom) coarse particulate organic matter (BCPOM; particles >1.0 mm), mostly composed of leaves and branches, was collected using a Hess sampler (0.09 m^2^). Attached algae was measured by scrubbing a 9-sq cm surface of randomly selected gravels and collecting the supernatent on glass fiber filters (pore size 1 μm, Whatman GF/B). Water samples were taken from the surface of river, poured through a sieve (1 mm) and then filtered onto glass fiber filters (1 μm). These filtered samples were used to quantify suspended fine particulate organic matter (SFPOM: 1 μm–1.0 mm), suspended solids (SS: 1 μm–1.0 mm) and chlorophyll-*a* (Chl-*a*) concentrations. BOM and POM were quantified by measuring ash free dry mass (AFDM; the decrease in mass of a sample after drying samples at 60°C for 24 h and combusting samples at 300°C for 30 min). Using the water samples, Biochemical Oxygen Demand (BOD), the amount of dissolved oxygen needed to biologically break down organic materials in the water sample, was measured as an index of organic pollution. Chl-*a* on the filters from the epilithon and water samples was quantified using a fluorescence spectrophotometer (HITACHI F-4500) following 99% methanol extraction (10°C, 24 h). All of the above biological variables were calculated as the mean of three replicate samples per site. Nutrient concentration in the water sample (dissolved inorganic nitrogen: NO_x_-N and NH_4_-N; soluble reactive phosphorus: PO_4_-P) were measured using a TRAACS 800 auto-analyzer (BLTEC, Japan) (*n* = 1).

### Species' niche and the specialization index (*S*)

The relative importance of each environmental variable in determining local habitat distribution was quantified using the specialization index (*S*) of Hirzel et al. [24]. *S* was calculated as the ratio of the standard deviation of an environmental variable across all sites (*σ*_g_) to the standard deviation of the variable at sites where the species is present (*σ*_s_) (*S* = *σ*_g_/*σ*_s_). When a species has an optimum for a given environmental variable, the variable is expected to show a narrow and nonrandom distribution at the sites where the species is present compared to the whole set of study sites. A randomly chosen set of sites is expected to have *S* = 1, with *S*>1 indicating some form of specialization of a species to that particular variable [24]. A high value of *S* indicates that a species occurs along only a narrow range of the total gradient available among all habitats.

### DNA extraction and AFLP fingerprinting

We genotyped 1793 individuals from 4 species (Table 1; 18.3 individuals/sites/species in average) using AFLP markers [27]. The digestive tract and epidermis were removed and DNA was isolated from remaining tissue using DNeasy 96 Blood and Tissue Kits (Qiagen, Tokyo). Genotyping was performed using the AFLP Plant Mapping Kit (Applied Biosystems, Tokyo) and the manufacturer's protocol with the following modifications: restriction was performed at 37°C for 3 h using 300 ng DNA with 1 U MseI, 5 U EcoRI, 5 μg BSA, 1 μL MseI buffer, 1 μL EcoRI buffer, and 13 μL sterilized dH_2_0. Ligation was then performed by adding 1 U T4 DNA ligase, 1 μL MseI adapter, 1 μL EcoRI adapter, 4 μL T4 DNA ligase buffer (10 x), and 14 μL sterilized dH_2_0 and incubating at 16°C for 10 h. Restriction/ligation products were then diluted 1: 5 with TE buffer (10 mM Tris-Hcl; 0.5 M EDTA) prior to pre-selective amplification using standard MseI and EcoRI oligonucleotide primers.

**Table 1:** Number of locations where species were present from among the 62 study sites, number of individuals used for AFLP analysis (*n*), number of outlier loci detected with the two methods applied (Dfdist with 95% significance level; BayeScan at posterior probability >0.95) and number of loci detected by both methods, and the number of neutral loci.

				Outlier loci			
Species	Sites	*n*	Total loci	BayeScan	Dfdist	both	Neutral loci
*Hydropsyche orientals* (Ho)	41	753	129	31	9	9 (7%)	98
*Stenopsyche marmorata* (Sm)	30	571	220	56	23	21 (10%)	164
*Hydropsyche albicephala* (Ha)	15	251	128	16	7	6 (5%)	111
*Ephemera japonica* (Ej)	12	218	473	23	7	7 (1%)	449

For the selective amplification reaction, 64 primer pairs contained in the AFLP Plant Mapping Kit were initially used to survey polymorphisms using three individuals of each species. The three primer pairs that generated the highest variability (Table S2 in File S1) were then used for all individuals. The three primer combinations yielded between 128 and 473 polymorphic loci in the four species (Table 1). Fluorescently labeled AFLP products (1 μl) were mixed with 0.1 μL ROX Size Standard (50–500 bp; Applied Biosystems) and 10 μL formamide and size-separated on an Applied Biosystems 3130 xl automated sequencer. GeneMapper v 2.1 (Applied Biosystems) was used to prepare a data matrix of the calculated sizes for all fragments from each individual. Peak intensity >200 RFU was used to determine the presence or absence of a fragment in the generated electropherogram. Scored fragments used for analysis ranged from 50–450 bp in length. Ten haphazardly chosen individuals of each species were analyzed twice (i.e., restriction, ligation, pre-amplification, selective amplification, size-separation, scoring) in order to test the repeatability of our AFLP methods. In these 40 individuals, 41 differences were observed out of 14670 scored fragments, indicating an error rate of 0.28%.

### Detection of outlier loci

Two different genome scan methods of Dfdist and BayeScan were applied to identify outlier loci. Dfdist is an extension of Fdist [28] allowing the use of dominant markers. This method uses coalescent simulations to generate thousands of loci evolving under a neutral model of symmetrical islands with a mean global *F*_ST_ among sampling sites close to the observed mean global *F*_ST_. Assuming that loci influenced by directional or balancing selection exhibit a larger or smaller genetic differentiation than neutral loci, the methods identify outlier loci that present observed mean global *F*_ST_ are significantly different from those expected under the neutral variation. Dfdist calculates observed global *F*_ST_ for dominant DNA markers at each locus using null-allele frequencies estimated by the Bayesian approach of Zhivotovsky [29]. Mean global *F*_ST_ was calculated using the default method of first excluding 30% of the highest and lowest observed values. Empirical loci with *F*_ST_ values significantly greater (*p*<0.05) than the simulated distribution (generated with 50,000 loci) were considered to be outliers.

An alternative approach, BayeScan, is a hierarchical Bayesian model-based method first described in [19], [22] for dominant markers. The Bayesian method is based on the idea that *F*_ST_ values reflect contributions from locus-specific effects such as selection, and population-specific effects such as local effective size and immigration rates. The main advantage of this approach is to allow different demographic scenarios and different amounts of genetic drift in each population. Using a reversible jump Markov chain Monte Carlo approach, the posterior probability that each locus is subject to selection is estimated. A locus is considered to be influenced by selection if its *F*_ST_ is significantly higher or lower than the expectation provided by the coalescent simulations. For all subsequent analyses, outlier loci were defined as loci detected by both methods at the 95% confidence level. Neutral loci were defined as loci detected by neither Dfdist nor BayeScan at the 95% thresholds. Loci detected as outliers by only one of the two methods were not considered in further analysis of either outlier or neutral loci. We further tested for pairwise linkage disequilibrium (LD) of the outlier loci detected by both methods, using 1000 steps in the Markov chain and a dememorization of 1000 steps in ARLEQUIN 3.5 [30].

In outlier detection, the possibility of detecting false positives (i.e. committing type-I errors), among the large number of screened loci is a general concern. For example, the number of loci expected to be outliers by random chance  = 0.05× number of loci (when a 95% criterion is employed). Correlation of allele frequencies across populations also potentially leads to a high rate of false positives because it invalidates the estimation of the variance of *F*_ST_ [31], [32]. Although the stochastic simulation approach [28] implemented in Dfsist and BayeScan improved the performance of the test, the correlations might be the case in our sampling design where high gene flow is expected among the geographically close sites. Especially, Dfdist is likely to generate false positives when gene flow is asymmetric across populations, and/or when some populations experience bottlenecks [33]–[35]. Therefore, a false discovery rate (FDR) of 10% was adopted for Dfdist analysis by using the program MCHEZA ([36]), which implements the method of [37]. BayeScan attempts to address the issue of detecting false positives by calculating a locus-specific effect in the overall pattern of genetic differentiation [19]. Therefore, the outliers conservatively defined in our study as loci detected by both methods including the Bayesian method are less likely to represent false positives.

### Analysis of genetic diversity and divergent selection

Three mean values of genetic differentiation (global *F*_ST_) among sampling sites were calculated for each species: for all loci, for only neutral loci, and for only non-neutral loci. Global heterozygosity (*H*_T_) and mean heterozygosity within sampling sites *(H*_W_) was estimated separately for neutral and non-neutral loci using a Bayesian approach with a uniform prior distribution of allele frequencies [29] implemented in AFLP-SURV v 1.0 [38].

**Figure 2:**
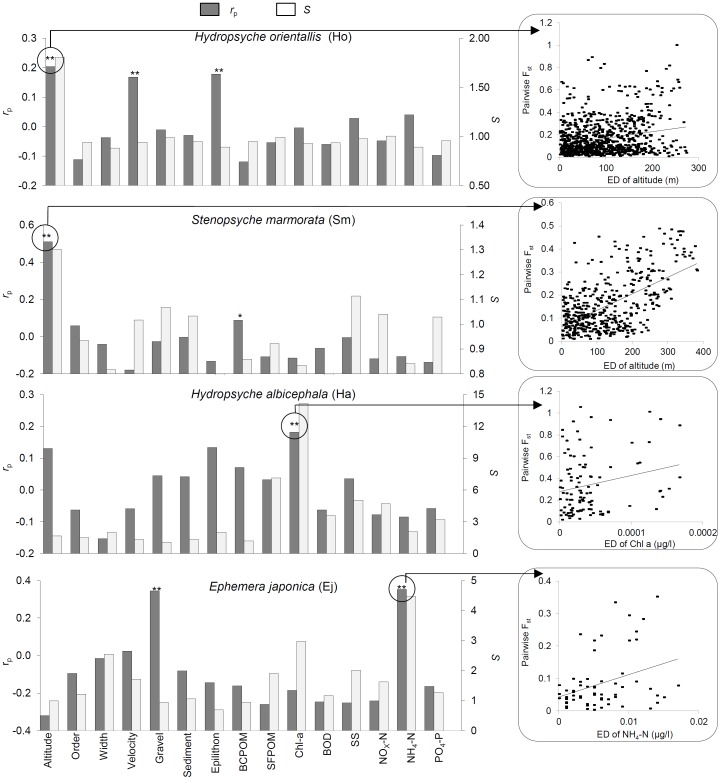
Specialization index (open bars) and genetic-environment correlation coefficients at outlier loci (filled bars) for each species and each environmental variable. Partial Mantel tests were used to calculate *r*_p_ in order to control for any effect of geographic distance. ** =  significant partial Mantel correlation (p<0.001, randomizations test). In each species, highest *r*_p_ and highest *S* occurred with the same environmental variable. These were altitude (Ho, Sm), transported chlorophyll-*a* (Ha), and ammonium-nitrogen (Ej). Correlations with highest *r_p_* are shown in the right-hand panels.

We used simple and partial Mantel tests [39] to test for correlation between pairwise genetic distances at non-neutral loci and environmental distance between sites. Some authors report that partial Mantel tests can result in incorrect p-values [40], [41]. Although several alternative methods based on the likelihood analysis [42] and mixed modelling approach [43], [44] have been proposed, the partial test remains a standard approach. Genetic distance between each pair of sites was quantified using the mean pairwise *F*_ST_ for all non-neutral loci. Each pairwise *F*_ST_ was calculated using the Bayesian-estimated allele frequencies generated by AFLP-SURV. The environmental difference between each pair of sites was quantified as a one-dimensional Euclidian distance for each variable (Fig. 2). Simple Mantel tests were used to examine patterns of Isolation By Environmental Distance (IBED; [45]). Because genetic isolation by geographical distance (IBD) was significant in three of the four species examined (Table S3), partial Mantel tests were carried out to control for geographic distance. All tests were implemented using 3000 randomizations and were conducted using a program written in C (available from the authors) that reports one-tailed probabilities.

## Results

### Genetic structure and outlier loci

Global *F*_ST_ among sampling sites of four study species (Fig. 1) ranged from 0.04–0.09 (Table 2) and standard errors overlapped 0.00 in each case (data not shown). Measured using only neutral loci (see below), *F*_ST_ was even lower (*F*_ST_ = 0.01–0.04; Table 2) throughout all species (Permutation test, *p*<0.0125). These results at neutral loci indicate high levels of gene flow in all of the species across the spatial scale of our study. Total genetic variance (*H*_T_) was similar in all species. Values were lower at neutral loci than at non-neutral loci (Table 2) and the same was true for mean genetic variance within sampling sites (*H*_W_).

**Table 2:** Summary of genetic structure and diversity measured using all loci, neutral loci, and outlier loci.

Statistic		Species		
	*Hydropsyche orientalis* (Ho)	*Stenopsyche marmorata* (Sm)	*Hydropsyche albicephala* (Ha)	*Ephemera japonica* (Ej)
*F*_ST_				
All loci	0.04 (.18)	0.07 (.14)	0.09 (.18)	0.04 (.28)
Neutral loci	0.00	0.03	0.03	0.02
Outlier loci	0.23	0.22	0.34	0.22
*H*_T_				
Neutral loci	0.11	0.11	0.12	0.07
Outlier loci	0.25	0.22	0.43	0.18
*H*_W_				
Neutral loci	0.11	0.12	0.12	0.07
Outlier loci	0.20	0.17	0.28	0.14

*F*_ST_ =  Wright's fixation index among sampling sites (with standard error in parentheses); *H*_T_ =  total expected heterozygosity within species; *H*_w_ =  mean expected heterozygosity within sampling sites.

Between 6 and 21 outlier loci were detected in the four species examined (Table 1). The proportion of outlier loci (using our criterion of significance with both methods) was not correlated to number of individuals used for AFLP analysis (*n*) or global *F*_ST_ (data not shown). Loci detected using BayeScan (Table 1) were under directional selection (α_i_>0). Between 11 (Ha) and 35 (Sm) loci were detected as outliers by only one of the two methods. These were removed from subsequent analysis because they could not be confidently considered outliers nor neutral. LD analysis found that 8–20% of possible pair-wise combinations of outlier loci (i.e. one of the outlier pairs in one of the sites) were statistically linked (Randomization test, *p*<0.01). The proportions of significant pairs were higher than expected by chance; however, there was no locus pair that was consistently in disequilibrium in multiple populations.

### Habitat specialization and adaptive divergence

Based on the highest significant values of the specialization index (*S*), species were most constrained by altitude (for Ho and Sm), algal concentration in the water (measured as chlorophyll-*a*)(for Ha), and ammonium-nitrogen (NH_4_) concentration in the water (for Ej)(Fig. 2)(Table S3). Other variables with large values of *S* included ammonium-nitrogen (Ho), water velocity (Ho), algal concentration (Ha), and substrate size (Ej).

At non-neutral loci, pairwise *F*_st_ among study sites were significantly correlated to between 1 and 3 environmental variables, depending on the species examined. This was based on partial Mantel tests of non-neutral *F*_ST_ and environmental (Euclidean) distance (Fig. 2, Table S3 in File S1). In all four species, the strongest correlation of pairwise genetic and environmental distances (i.e., highest partial correlation coefficient (*r*_p_)) was with the variable most limiting species distributions (i.e., highest *S*). Specifically, pairwise *F*_st_ at non-neutral loci were most closely related to changes in altitude (Ho, Sm), transported chl-*a* (Ha), and ammonium-nitrogen (Ej) (Fig. 2). With 15 environmental variables, the probability that the variable with highest *S* also has the highest *r*_p_ for the 4 species was significantly higher than would be expected by chance (*p*<0.01, Fisher's combined probability test). Partial mantel correlations for the other significant variables were also significant in each case, but lower (Fig. 2).

## Discussion

### Adaptive genetic divergence along narrow environmental gradients

There is a broad range of evidence for adaptive divergence of populations [7]–[9], and techniques are now available for the study of the genetic signatures of adaptive divergence in non-model organisms (e.g., [14], [15]). An important next step in our understanding of adaptive divergence is to identify general patterns by examining multiple species and environmental characteristics simultaneously in nature. Our anonymous marker approach applied to four co-occurring species and a range of environmental characteristics is complementary to studies that test whether one or a few *a priori* loci or phenotypic traits are under selection in single species (e.g., [14], [16], [17]). We used genome scans (AFLP) to detect outlier loci presumably experiencing adaptive divergence. We then used Mantel test to identify several environmental factors contributing to the divergence at these loci. We used a “reverse ecology” approach (*sensu* [46]), whereby a suite of habitat and resource components of the niche were measured and correlation was applied to identify those driving adaptive divergence. We uncovered strong evidence for adaptive divergence in these riverine insects, and by simultaneously studying four species, we identified a striking trend: the environmental variables that were most significantly associated with genetic distance at non-neutral loci were the same variables that limit the species geographical distribution, as measured using the specialization index (*S*) of Hirzel et al. [47].

Adaptive divergence thus appears to be driven by the narrowest ecological gradient in each species, i.e. the most restricted spectrum of possible environments (*sensu* [48]). That the steepest ecological gradient is also the narrowest is not necessarily obvious, and many studies of adaptive divergence focus on the extremes of a particular environmental factor that occur over broad scales such as temperature extremes across broad latitudinal [16] and elevational [17] gradients. Nonetheless, if a niche is narrow, then genetic change (i.e., adaptive divergence) may be required to accommodate even a small environmental distance regardless of how steep a particular gradient may appear in our observations. Maladaptive gene flow from central to peripheral populations of a species' range (“migration load”; [49]–[51]) may account for the observed patterns. The immigrant genes may be adapted to the range center and thus inhibit adaptation at the periphery [52], making a species' range a function of gene flow and the steepness of a given selection gradient. What is interesting in our findings is that the species studied occur throughout Japan, thus while a strict range-margin explanation seems unlikely, it may be that similar processes operate between locally adapted populations on relatively small spatial scales. Another explanation is an intrinsic limit of species' evolutionary capacity [53]. The low frequency of mutations may not allow peripheral populations to adapt to greater environmental extremes. Finally, an intriguing alternative explanation is interspecific competition for space or resources. In theory [54], populations tend to diverge along the narrowest niche dimension because the dimension still has a space to drive the populations to use alternative resources, which provide a greater chance to escape from the high competition however this process is determined by the balance between the inherent costs and benefits of widening the niche [55]. We do not have any measures of competition to test this hypothesis, although local population densities can be very high in these species.

Before drawing firm conclusions about any general role of the particular environmental gradients identified here, it is important to consider both the characteristics themselves and the temporal scale of measurement. Altitude was most strongly correlated with non-neutral pairwise *F*_st_ in two species (Ho and Sm). Altitude itself is related to a number of resource and habitat variables in stream ecosystems, some of which were measured here (e.g., stream size, water velocity), and some of which were not measured (solar insolation, primary production). Temperature is often an *a priori* factor for which adaptation is tested (e.g., [17]) because of its overwhelming influence on organismal physiology. In stream environments like those studied here, altitude is often strongly associated with mean annual water temperature, and the stronger genetic selection for altitude compared to temperature may well reflect the fact that altitude is a better proxy for complex differences in thermal regime than our two point measurements in the course of one year. Species' distributions are often constrained by extreme thermal conditions (i.e. maxima or minima) rather than mean conditions, therefore, in future study, further analysis using more detailed data such as time-series thermal measures may be feasible. For example, there is experimental evidence for thermal limits in the two species that showed strongest adaptation along the altitudinal gradient. Ho cannot oviposit in air temperatures lower than 11°C (unpublished data) and Sm cannot pupate or emerge in water temperatures lower than 11°C and 13°C, respectively [56].

Ammonium-nitrogen and chlorophyll-*a* that reflect benthic and planktonic algae also exhibited clear relationships with pairwise *F*_st_. Like altitude, nitrogen may act as a general indicator of environmental pollution and habitat degradation. The sensitivity of mayflies to such factors is well documented in biomonitoring studies [57]. The link with chlorophyll-*a* in the water column is less clear, but this can be influenced by benthic algae that has been dislodged from the stream bottom or planktonic algae that has been transported downstream from standing water zones. Both are important components of the diet of filter-feeding Trichoptera. Other unmeasured factors such as disease [58] or predation [59] also may play a role in adaptive divergence.

The observed genetic divergences at loci under selection were maintained despite relatively high levels of gene flow observed at neutral loci. This is in contrast to previous studies that were conducted at larger spatial scales and reported some level of neutral population differentiation (e.g., [14], [16]) and indicates that selection pressures are strong enough to counter homogenization by migration [60]. The fact that species showed little or no population differentiation at neutral loci is similar to what has been observed in other riverine caddisflies at the spatial scale examined here (up to ca. 50 km; see [61], [62]) although it is risky to attempt a generalization across species [63]. Individuals are therefore able to disperse widely, with local conditions determining the genetic makeup of those individuals who persist.

### Genetic structure using neutral and non-neutral loci

By comparing neutral and non-neutral genetic differentiation, we can draw some inferences about the process of genetic divergence in natural populations in a way analogous to *F*_ST_ - *Q*_ST_ comparisons, where *F*_ST_ represents neutral variation and *Q*_ST_ represents variation in quantitative trait such as morphology [64]. Similar selection pressures (i.e., stabilizing selection) acting throughout the range of sites should lead to lower differentiation at non-neutral loci compared to neutral loci which are primarily governed by genetic drift. We did not observe this pattern, but found the opposite pattern of diversifying selection showing higher non-neutral differentiation than neutral differentiation. Several empirical studies for animals such as snails [65], spiders [66], and damselflies [67]–[69] compared levels of divergence between morphological traits (analogous to non-neutral markers) and neutral molecular markers. Most of these studies support our results, showing greater morphological divergence than differentiation as measured through *F*_ST_ based on neutral markers. However, one damselfly study observed the opposite pattern [70], and another damselfly study reported a temporal shift of the pattern from stabilizing selection (lower morphological divergence) to diversifying selection over the course of two generations [71].

Genetic diversity was higher when measured with non-neutral loci compared to neutral loci, suggesting that adaptive divergence is an important driver of total genetic diversity at this spatial scale [72]. The greater diversity within subpopulations at non-neutral loci supports a theory of migration-selection balance which predicts that, as long as adaptive divergence is strong on the spatial scale at which gene flow occurs [73]–[75], diversity is increased through mixing of diverse alleles from different subpopulations. One shortcoming of our study design is that selection that occurs within individual sampling sites (i.e., along microhabitat gradients) would be obscured because our analysis of alleles and environmental variables was at the site-scale. Thus while our results provide clear evidence of adaptive divergence among different stream reaches (ca. 300 m long), it may also be that within-site, micro-habitat scale divergence also contributes to total genetic diversity. From a conservation standpoint, our results suggest that ecologically marginal habitats, for example those that may occur at the edge of a species' range of niche, harbor genetic diversity that is potentially important for adaptive diversification. When marginal habitats are lost, rare genotypes adapted to these margins will be lost.

## Supporting Information

File S1 Table S1, the 31 environmental variables measured at each site and Pearson's correlation coefficients between them. Table S2, oligonucleotide primer pairs used for selective amplification (AFLP PCR). Table S3, characteristics of the 15 non-redundant environmental variables and their relationship to allele frequency at outlier loci.
(DOCX)
