# Supplementary material for: Adaptive Genetic Divergence along Narrow Environmental Gradients in Four Stream Insects
Source: PLoS One. 2014 Mar 28;9(3):e93055. doi: 10.1371/journal.pone.0093055 (PMC3969376; doi:10.1371/journal.pone.0093055)
Supplement: File S1 — Table S1, the 31 environmental variables measured at each site and Pearson's correlation coefficients between them. Table S2, oligonucleotide primer pairs used for selective amplification (AFLP PCR). Table S3, characteristics of the 15 non-redundant environmental variables and their relationship to allele frequency at outlier loci. (DOCX) [file pone.0093055.s001.docx]

**Table S1. The 31 environmental variables measured at each site.** When multiple variables are listed on a single line, only the first was used for analysis. Pearson's correlation coefficients (*r*) of the analyzed variables with the other variables are given in parentheses. See the main text (Environmental variables section in Methods) for the description of the analyzed variables in the first column.

| Analyzed variables (*n* = 15) | Other variables (*n* = 16) |
| --- | --- |
| Altitude (m) | Distance from river mouth (0.90); % Vegetation^‡^ (0.62); pH (-0.51); % Urban^‡^ (-0.40); % Farm^‡^ (-0.34) |
| Stream order | Mean water depth at riffles (0.69) |
| Channel width (m) | - |
| Current Velocity (m/s) | Latitude (0.30) |
| Gravel size (cm) | - |
| Sediment size (mm) | Curvature Coefficient of sediment grain size distribution (0.37) |
| Epilithon (mg Chl-a/cm^2^) | AFDM^§^ of epilithon (0.60) |
| BCPOM^*^ (mg AFDM^§^/m^2^) | AFDM^§^ of Benthic Fine Particulate Organic Matters (0.37); Longitude (0.24) |
| SFPOM^†^(μg AFDM^§^/L) | Uniformity Coefficient ofof sediment grain size distribution (0.31) |
| Chlorophyll-*a* (μg/L) | Standard Deviation of gravel size (0.47) |
| BOD (mg/L) | - |
| Suspended Solid (mg/L) | - |
| NO_X_-N (μg/L) | Total Inorganic Nitrogen concentration in river water (1.00); Electric Conductivity (0.34) |
| NH_4_-N (μg/L) | NO_2_-N concentration in river water (0.86) |
| PO_4_-P (μg/L) | - |

^*^Benthic Coarse Particulate Organic Matters

^†^Suspended Fine Particulate Organic Matters

^‡^ % Vegetation, % Urban, and % Farm are area ratio of the land use category quantified through grid data (50 m resolution) of the Digital National Land Information database of the Miyagi prefecture by Ministry of Land, Infrastructure, Transport and Tourism (MLIT), Japan.

^§^Ash Free Dry Mass

**Table S2. Additional nucleotides on oligo primer pairs used for selective amplification (AFLP PCR).**

| Primer pair | EcoRI+ | MseI+ |
| --- | --- | --- |
| 1 | ACA | CAC |
| 2 | ACG | CTT |
| 3 | AAC | CAG |

**Table S3. Characteristics of the 15 non-redundant environmental variables and their relationship to allele frequency at outlier loci.** Summary of the 2 indices on the ecological niche space (% and *S*) for 4 species regarding to 15 environmental variables, and the results of simple (*r*) and partial (*r*_p_) Mantel tests between genetic and environmental distances. The full range of environment represent minimum and maximum values among a whole set of study sites (*n*=62). % is the percentage of environmental range among sites where species occurred (*% range*) and *S* is the specialization index. Partial Mantel tests were performed controlling for geographic distance. Bolds values indicate the lowest (%) and highest (*S*, *r*, *r*_p_) observed for each species.

|  |  |  |  |  | *Hydropsyche orientallis* | | | |  | *Stenopsyche marmorata* | | | |  | *Hydropsyche albicephala* | | | |  | *Ephemera japonica* | | | |
| --- | --- | --- | --- | --- | --- | --- | --- | --- | --- | --- | --- | --- | --- | --- | --- | --- | --- | --- | --- | --- | --- | --- | --- |
|  | Full range | | |  | % | *S* | *r* | *r*_p_ |  | % | *S* | *r* | *r*_p_ |  | % | *S* | *r* | *r*_p_ |  | % | *S* | *r* | *r*_p_ |
| Altitude (m) | 2 | - | 590 |  | **46** | **1.81** | **0.24**^**^ | **0.20**^**^ |  | **65** | **1.30** | **0.58**^**^ | **0.51**^**^ |  | 55 | 1.65 | 0.16 | 0.13 |  | 79 | 0.99 | -0.21 | -0.32 |
| Order | 1 | - | 5 |  | 100 | 0.94 | -0.12 | -0.11 |  | 100 | 0.93 | 0.03 | 0.06 |  | 50 | 1.50 | -0.08 | -0.06 |  | 75 | 1.22 | -0.10 | -0.09 |
| Width (m) | 1 | - | 38 |  | 100 | 0.88 | -0.05 | -0.04 |  | 100 | 0.82 | -0.08 | -0.04 |  | 34 | 1.97 | -0.16 | -0.15 |  | 28 | 2.54 | -0.02 | -0.02 |
| Velocity (m/s) | 0.03 | - | 1.29 |  | 100 | 0.94 | 0.18^**^ | 0.17^**^ |  | 83 | 1.02 | -0.18 | -0.18 |  | 49 | 1.32 | -0.11 | -0.06 |  | 42 | 1.70 | 0.01 | 0.02 |
| Gravel size (cm) | 5 | - | 23 |  | 89 | 0.99 | -0.02 | -0.01 |  | 78 | 1.07 | -0.03 | -0.03 |  | 75 | 1.05 | 0.06 | 0.04 |  | 84 | 0.94 | 0.33^**^ | 0.34^**^ |
| Sediment size (mm) | 0.5 | - | 19.0 |  | 100 | 0.95 | -0.03 | -0.03 |  | 100 | 1.03 | -0.03 | 0.00 |  | 63 | 1.32 | 0.05 | 0.04 |  | 79 | 1.05 | -0.09 | -0.08 |
| Epilithon (mg AFDM/cm^2^) | 0.01 | - | 13.07 |  | 100 | 0.89 | 0.20^**^ | 0.18^**^ |  | 100 | 0.79 | -0.08 | -0.13 |  | 37 | 2.01 | 0.15 | 0.13 |  | 99 | 0.68 | -0.12 | -0.14 |
| BCPOM (mg AFDM/m^2^) | 0.90 | - | 54.07 |  | 100 | 0.95 | -0.11 | -0.12 |  | 98 | 0.86 | 0.08 | 0.09 |  | 46 | 1.18 | 0.12 | 0.07 |  | 67 | 0.94 | -0.11 | -0.16 |
| SFPOM(μg AFDM/L) | 0.9 | - | 23.7 |  | 100 | 0.99 | -0.02 | -0.05 |  | 100 | 0.92 | -0.03 | -0.11 |  | 12 | 7.14 | 0.02 | 0.03 |  | 41 | 1.90 | -0.24 | -0.26 |
| Chl-a (μg/L) | 0.06 | - | 2.33 |  | 100 | 0.93 | -0.01 | 0.00 |  | 100 | 0.83 | -0.13 | -0.12 |  | **7** | **14.16** | **0.22**^**^ | **0.18**^*^ |  | 30 | 2.97 | -0.17 | -0.19 |
| BOD(mg/L) | 0.0 | - | 2.4 |  | 100 | 0.93 | -0.05 | -0.06 |  | 100 | 0.80 | -0.02 | -0.06 |  | 18 | 3.57 | -0.06 | -0.06 |  | 60 | 1.17 | -0.25 | -0.25 |
| Suspended Solid (mg/L) | 0.3 | - | 11.8 |  | 98 | 0.98 | 0.03 | 0.03 |  | 83 | 1.11 | 0.05 | -0.01 |  | 18 | 5.03 | 0.09 | 0.04 |  | 25 | 2.01 | -0.19 | -0.25 |
| NO_X_-N (μg/L) | 0.06 | - | 1.51 |  | 93 | 1.01 | -0.03 | -0.05 |  | 91 | 1.04 | -0.04 | -0.12 |  | 21 | 4.71 | -0.03 | -0.08 |  | 56 | 1.62 | -0.17 | -0.24 |
| NH_4_-N (μg/L) | 0.004 | - | 0.147 |  | 99 | 0.89 | 0.05 | 0.04 |  | 99 | 0.84 | -0.08 | -0.11 |  | 31 | 2.06 | -0.09 | -0.09 |  | **12** | **4.47** | **0.35**^**^ | **0.35**^**^ |
| PO_4_-P (μg/L) | 0.001 | - | 0.047 |  | 99 | 0.96 | -0.07 | -0.10 |  | 93 | 1.03 | -0.09 | -0.14 |  | 28 | 3.18 | -0.05 | -0.06 |  | 49 | 1.26 | -0.14 | -0.16 |
| Geo. Distance (km) | 1.4 | - | 42.8 |  |  |  | 0.13^**^ |  |  |  |  | 0.33^**^ |  |  |  |  | 0.21^**^ |  |  |  |  | 0.07 |  |
| Mean |  |  |  |  | 95 | 1.00 |  |  |  | 93 | 0.96 |  |  |  | 36 | 3.46 |  |  |  | 55 | 1.70 |  |  |

*= *p*  < 0.05; ** = *p* < 0.01
